# Mapping the genomic mosaic of two ‘Afro-Bolivians’ from the isolated Yungas valleys

**DOI:** 10.1186/s12864-016-2520-x

**Published:** 2016-03-09

**Authors:** Jacobo Pardo-Seco, Tanja Heinz, Patricia Taboada-Echalar, Federico Martinón-Torres, Antonio Salas

**Affiliations:** Unidade de Xenética, Departamento de Anatomía Patolóxica e Ciencias Forenses, and Instituto de Ciencias Forenses, Grupo de Medicina Xenómica (GMX), Facultade de Medicina, Universidade de Santiago de Compostela, Calle San Francisco s/n, C.P. 15872, Galicia, Spain; Grupo de Investigación en Genética, Vacunas, Infecciones y Pediatría (GENVIP), Hospital Clínico Universitario and Universidade de Santiago de Compostela (USC), Galicia, Spain; Infectious Diseases and Vaccines Unit, Department of Pediatrics, Hospital Clínico Universitario de Santiago, Santiago de Compostela, Galicia Spain

**Keywords:** Transatlantic slave trade, Afro-Bolivians, Ancestry, Genome, SNPs

## Abstract

**Background:**

Unraveling the ancestry of ‘Afro-American’ communities is hampered by the complex demographic processes that took place during the Transatlantic Slave Trade (TAST) and the (post-)colonization periods. ‘Afro-Bolivians’ from the subtropical Yungas valleys constitute small and isolated communities that live surrounded by the predominant Native American community of Bolivia. By genotyping >580,000 SNPs in two ‘Afro-Bolivians’, and comparing these genomic profiles with data compiled from more than 57 African groups and other reference ancestral populations (*n* = 1,161 in total), we aimed to disentangle the complex admixture processes undergone by ‘Afro-Bolivians’.

**Results:**

The data indicate that these two genomes constitute a complex mosaic of ancestries that is approximately 80 % of recent African origin; the remaining ~20 % being European and Native American. West-Central Africa contributed most of the African ancestry to ‘Afro-Bolivians’, and this component is related to populations living along the Atlantic coast (i.e. Senegal, Ghana, Nigeria). Using tract length distribution of genomic segments attributable to distinct ancestries, we could date the time of admixture in about 400 years ago. This time coincides with the maximum importation of slaves to Bolivia to compensate the diminishing indigenous labor force needed for the development of the National Mint of Potosí.

**Conclusions:**

Overall, the data indicate that the genome of ‘Afro-Bolivians’ was shaped by a complex process of admixture occurring in America among individuals originating in different West-Central African populations; their genomic mosaics received additional contributions of Europeans and local Native Americans (e.g. Aymaras).

**Electronic supplementary material:**

The online version of this article (doi:10.1186/s12864-016-2520-x) contains supplementary material, which is available to authorized users.

## Background

During the Transatlantic Slave Trade (TAST) from the fifteenth to the end of the nineteenth century, more than 12.5 million enslaved Africans boarded ships that were destined to the Americas. Most African slaves brought to the Americas came from West-Central Africa (~5.6 million) [1]. African slaves were not distributed uniformly in the American regions of interest. In Brazil, for example, most Africans were forced to migrate to the Northeastern part of the country, that is, to Pernambuco and Bahia, because this region developed to become one of the most important sugar production zones during the TAST period [2]. Similarly, in Colombia there was high demand for experienced gold miners especially in the department of Chocó [3].

During the last decade, geneticists have aimed to unravel the complex patterns of admixture occurring in America as a consequence of the European colonization and the TAST. In particular, the analysis of mitochondrial DNA (mtDNA) has been widely used in the literature [4–10]. For instance, it is now known that African mtDNA lineages in America prevailed in the Northeast of Brazil [11], and are also widely distributed in ‘Afro-Colombians’ of Chocó [12–14]. According to autosomal DNA, many populations in Brazil are principally of European ancestry, but there exists a North–South gradient towards an increased African ancestry in the North, Northeast, and Centre-West [15]. Similarly, in Colombia, a pronounced autosomal African admixture was observed in individuals from Chocó [16]. Moreno-Estrada et al. [17] investigated the population genetic history of the Caribbean by characterizing patterns of genome-wide variation. These authors found that admixed genomes can be traced back to distinct sub-continental source populations, even in situations where limited pre-Columbian Caribbean haplotypes survived.

Very little attention has so far been given to historically isolated populations whose history can also be traced back to the TAST, as is the case of the ‘Afro-Bolivian’ community of Tocaña, located in the Nor (North) and the Sud (South) Yungas valleys of Bolivia. The Yungas are located in the Department of La Paz, in the passageway that connects the Bolivian highlands and the tropics. During the colonial period, Spaniards initially used indigenous people as a workforce to exploit the region’s mineral wealth at the mines and Mint of Potosí, but soon they began to enslave Africans. Mortality rate of slaves was high in the region mainly due to the fact that they were forced to work in a region over 4,000 m above sea level and in very hard conditions [18]. The first African slaves in Potosí were recorded beginning in 1549 [19]. Upper Peru, nowadays Bolivia, received a considerably number of African enslaved people at the beginning of the TAST from Senegambia; however, West-Central Africa became increasingly important at the end of the sixteenth century. Rodriguez [18] also mentions that at least 1,536 people were brought to Bolivia from the Congo-Angola area and Mozambique. The region around present-day Angola might also have been an important source of enslaved people for Spanish traders, because when the Spanish and Portuguese crown merged between 1580 and 1640, many enslaved Africans from the hinterlands of Luanda in Angola, which was kept by Portugal, were forced to migrate to Spanish America [2, 20].

African enslaved people from West-Central Africa principally arrived in Upper Peru *via* Río de la Plata, and at least between 1,500 and 3,000 Angolan enslaved people arrived at Río de la Plata every year during the first half of the seventeenth century [20]. It is also documented that in 1807, there were 458 Africans in Potosí to work in the mint [21]. Later, Spanish colonists began to use slaves in agricultural work in the tropical valleys. Rodriguez [18] reported that, in 1883, the enslaved ‘black’ population in the region consisted of more than 6,000 people.

Although ‘Afro-Bolivians’ were not included in the official National Census of 1996, it was estimated at that time that about 10,000 ‘Afro-Bolivians’ were mostly concentrated in the Yungas provinces, mainly in rural towns and villages such as Coroico, Irupana, Tocaña, etc. This small community adopted much of the technological and economic organization and cultural norms of the local indigenous Aymara [18]. Only very recently, the reclaiming of a ‘Afro-Bolivian’ culture has begun to emerge in the region by the creation of cultural organizations aimed to recover their lost cultural identities.

Some inferences on their origins have been made based on linguistics [22]. There are unique words that probably derived from Kikongo, a language spoken in Congo. Furthermore, there exist two common African surnames in ‘Afro-Bolivian’ communities, Angola and Maconde, the latter of which might also be of Congolese origin [22].

‘Afro-Bolivian’ groups have hitherto received very little attention within the scientific community that investigates the history of the TAST; probably because they constitute a relatively small community surrounded by a numerous Aymara-speaking population, and because they live in a geographically remote region. Similarly, genetics research on Bolivian populations has primarily focused on the Native American indigenous population excluding people of African ancestry. So far, mtDNA lineages in the departments of Beni, Chuquisaca, Cochabamba, La Paz, Pando, and Santa Cruz, which are distributed across three eco-geographically distinct regions (Andean, Sub-Andean, Llanos), have been shown to be mainly of Native American ancestry [23–28]. In contrast, the Y-chromosome shows an important contribution of European colonizers [28–31]. Likewise, autosomal DNA analysis (mainly carried out using small panels of autosomal SNPs) shows a main Native American ancestry, although with an increased European introgression [23, 24]. In contrast, African ancestry was observed to be marginal in both mtDNA and autosomal DNA, respectively [23, 24, 32]. Only the community of Tocaña (Nor Yungas) still preserves the African genetic legacy of the TAST [33] as inferred from the uniparental markers and a few ancestry informative (autosomal) markers (AIMs).

The aim of the present study is to provide a first insight into the complex admixture processes experienced by individuals belonging to the geographically isolated ‘Afro-Bolivian’ community. In contrast to other ‘Afro-American’ communities that admixed in complex demographic circumstances (e.g. in the USA, Caribbean, Colombia, Brazil, etc.), ‘Afro-Bolivians’ from the Yungas valleys constitute very isolated communities since their initial formation, and have remained surrounded by peoples of main Native American ancestry. These ‘Afro-Bolivians’ therefore constitute a sort of ‘genetic laboratory’ to gain new insight into the TAST.

## Results

### Analysis of identity-by-state and multidimensional scaling analysis

The genetic proximity of the two Tocaña profiles to different sub-Saharan African groups can be studied by examining the genetic distances in terms of Identity-by-State (IBS) values between these two Bolivians and each of the population sample sets in Africa. These analyses were carried out using separately the population sets from The 1000 Genomes Project (hereafter 1000G) and a large dataset of African populations (including those that contributed more slaves to the TAST). The exploratory analysis carried out with 1000G samples (involving >500 K SNPs; see Material and Methods for details and Additional file 1 for the full list of population samples used for comparison) demonstrates that the two Bolivians have the highest IBS values with Africans (represented here by West-Central and East Africa), and the lowest values with non-Africans (Additional file 2A). The second round of analysis (Additional file 2B; 25 K SNPs), using a panel of 57 African datasets, indicates that the highest values of IBS for the two Tocaña are with the Yoruba (Nigeria); followed by a set of populations that are mainly from West-Central Africa. The lowest IBS values are between the two Bolivians and North Africans.

In order to better visualize the population relationships between the two ‘Afro-Bolivians’ and the main continental groups, a MDS was carried out with the main continental groups represented in 1000G. Dimension 1 and Dimension 2 (Additional file 3; >500 K SNPs) clearly separate sub-Saharans, East Asians, and Europeans in three tight clusters, each forming the vertex of an equilateral triangle. The remaining individuals (many of them admixed) plot in intermediate positions between the European edge and the other two clusters, in good agreement with their documented admixed ancestry; e.g. Puerto Rico shows a main projection from the European pole towards the African one, suggesting African admixture. In this scenario of continental ancestries, the two ‘Afro-Bolivians’ clearly show a close proximity to the sub-Saharans, in both the Dimension 1 and the Dimension 2, with a minor projection towards the other two vertexes of the triangle.

Once the predominantly African nature of the two ‘Afro-Bolivians’ was revealed, a second round of MDS was carried out in order to investigate the relationships of the two Bolivians with different African sub-continental regions. The plot of Dimension 1 and Dimension 2 shown in Additional file 4 (25 K SNPs) again displays a triangular arrangement, with the vertexes attracting South Africans (although very dispersed in the triangle denoting admixture with other African groups), North Africans (with clear affinities to Europeans; here represented by CEU), and West-Central Africans. East Africans scatter along the side of the triangle that connects West-Central Africans to North Africans. South-East Africans plot very close to the vertex occupied by the West-Central African samples with some projection into the South and East African poles as corresponds with their admixed history [10]. The two individuals from Tocaña locate in between the West-Central pole and to East Africans (Additional file 4).

**Fig. 1:**
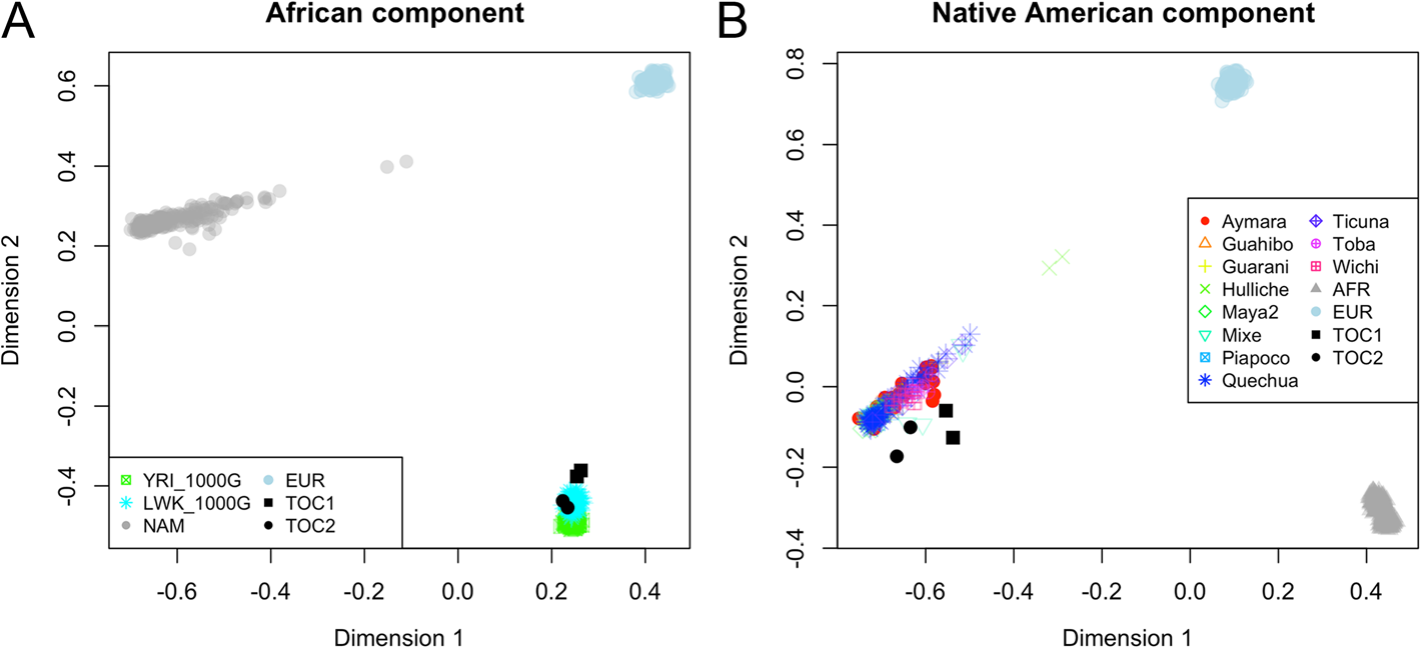
PCAmask based on 99 K SNPs focused on the Native American (**a**) and the African ancestry (**b**) of the two Tocaña

Finally, PCAmask was run on a reduced set of population samples. The first two dimensions (Fig. 1) indicate that the African ancestry of the Tocaña haplotypes is closely related to the main African group formed by the Yoruba and the Luhya. The Native American component of the two Tocaña falls very close to the main Native American pole that includes the Aymara and the Quechua.

### Disentangling the African component of ‘Afro-Bolivians’

**Fig. 2:**
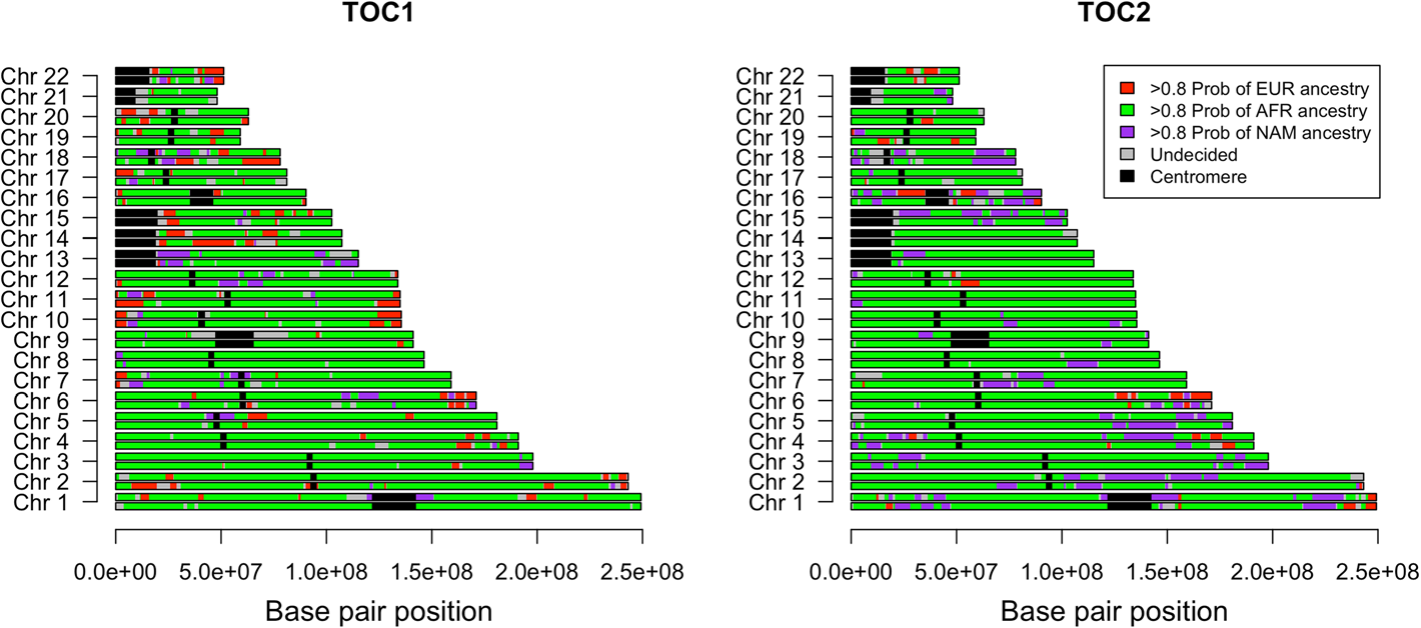
PCAdmix indicating the genomic architecture of the Tocaña individuals from the point of view of their main continental ancestries

PCAdmix was performed in order to reveal the genome architecture of the two Afro-Bolivians analyzed (Fig. 2). The analysis (>99 K SNPs) confirms the predominantly African component suggested by the MDS and indicates a disperse genomic pattern for the European and Native American components, in good agreement with the historical process of admixture and incompatible with modern events of admixture. Percentages of admixture in the two Tocaña calculated using PCAdmix were: (*i*) African ancestry: 78.1 for #TOC1 and 82.1 % for #TOC2; (*ii*) European ancestry: 14.8 for #TOC1 and 2.6 % for #TOC2; and (*iii*) Native American ancestry: 7.1 for #TOC1 and 15.3 % for #TOC2.

**Fig. 3:**
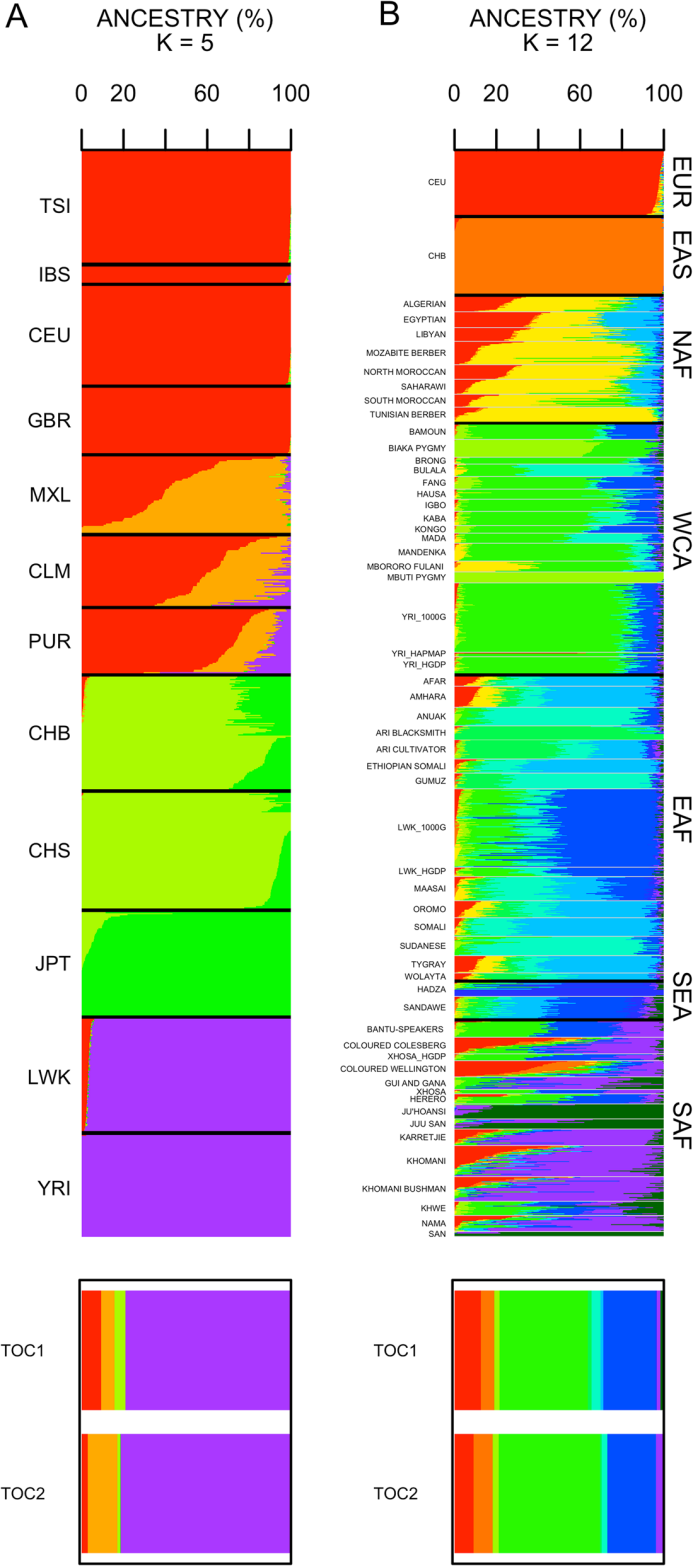
Bar-plot of individual ancestries as computed using the unsupervised clustering algorithm implemented in ADMIXTURE. **a** considers the populations in 1000G (*K* = 4 was the lowest cross validation value). **b** considers a wide set of African populations that represent main sub-continental regions; one European [CEU] and one East Asian [CHB] sample were included for reference (*K* = 12 was the lowest cross validation value)

An initial analysis performed with ADMIXTURE was carried out between the two Yungas individuals and the population sets from 1000G (>500 K SNPs). For an optimum *K* = 5 (see Additional file 5 for other *K* values), this analysis (Fig. 3a) confirms that the two Bolivians have a main African ancestry (#TOC1: 79.1 %; #TOC2: 81.4 %; Additional file 6). Furthermore, according to this analysis, both Bolivians have more Native American (#TOC1: 11.6 %; #TOC2: 15.7 %) than European (#TOC1: 9.3 %; #TOC2: 2.9 %).

Therefore, even though different reference population datasets and algorithms were used, percentages of admixtures obtained using PCAdmix and ADMIXTURE are broadly comparable for the three ancestral components. Both Tocaña have a similar proportion of African ancestry (79–81 %); the main difference between them is that #TOC2 has a larger proportion of Native American ancestry than #TOC1, which is balanced by a lower European ancestry in #TOC2.

Analysis of admixture was also carried out with the large set of African populations (>25 K SNPs) and the optimum *K* = 12 (Fig. 3b; Additional file 7; see Additional file 8 for other *K* values). Some findings of this exploratory analysis are suggestive. First, it indicates that the two Bolivians have one predominant component (42–49 %) that coincides with the predominant component in West-Central Africa (56 on average); in particular, in the Yoruba (Nigeria; 77), Hausa (Cameroon; 71), Brong (Ghana; 79), and Mandenka (Senegal; 77 %); Additional file 7. The second most important component in the two Tocaña (23–25 %) is shared with a subset of East Africans; out of the whole African dataset this component reaches the maximum values in the Luhya (46–55 %; Additional file 7).

### Disentangling the Native-American component of ‘Afro-Bolivians’

**Fig. 4:**
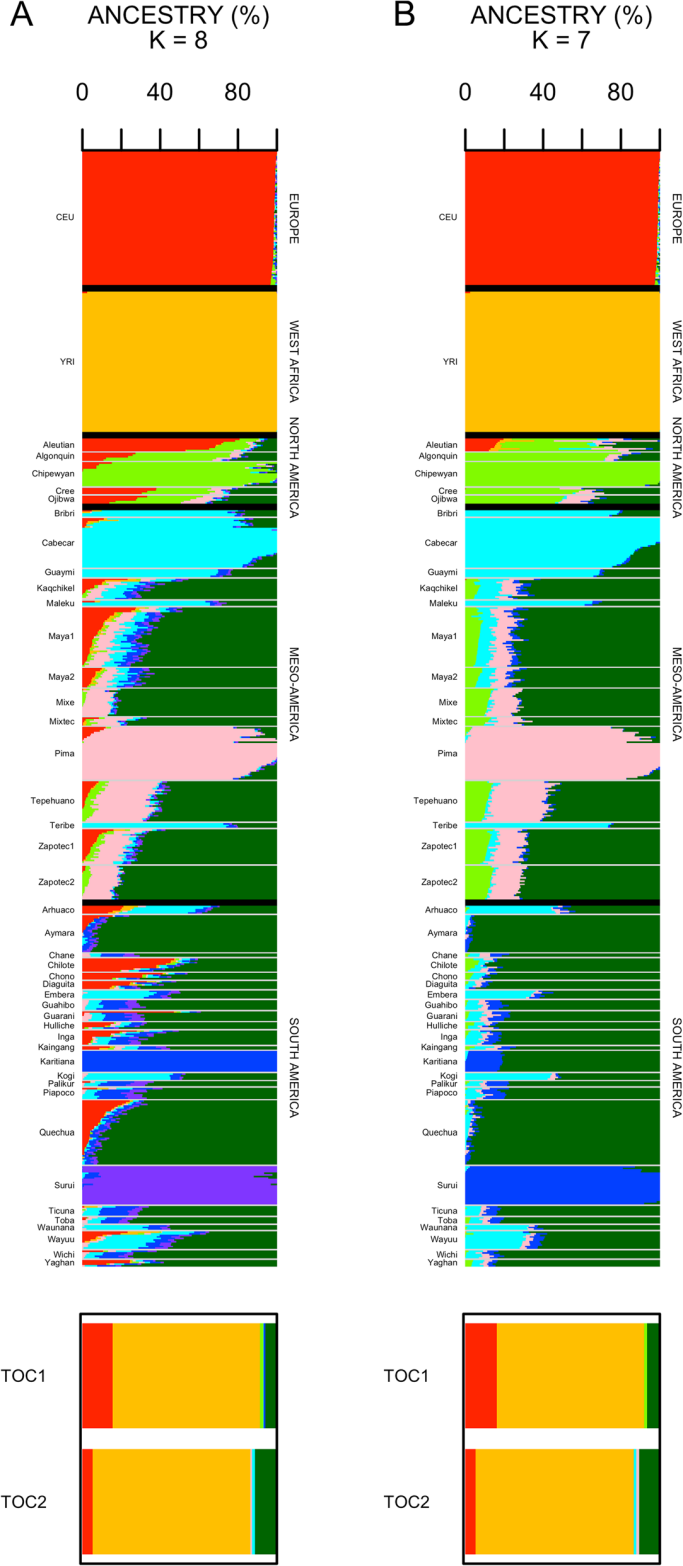
Analysis of admixture of the two Tocaña individuals with unmasked (**a**) and masked (**b**) Native American datasets

Additional analyses were carried out in order to further investigate the Native American component of ‘Afro-Bolivians’ as suggested by ADMIXTURE and PCAdmix. This analysis is somehow blurred by: (*i*) the fact that all the Native American groups used as reference populations are also admixed to different extents with Europeans and even with people of recent African ancestry [34], and (*ii*) the complex admixture nature of the two Tocaña individuals investigated. The analysis was carried out using the unmasked (using the whole reference datasets; >99 K SNPs) and the masked (filtering out the non-Native American component of the individuals in the reference populations; >92 K SNPs) data of the Native American groups analyzed by Reich et al. [34]; Fig. 4.

ADMIXTURE reveals the presence of a unique Native American component in the Tocaña, and a pattern that is also present in the sub-Andean/Andean Bolivian populations (represented e.g. by Aymaras). Also remarkable is the fact that the Native American component of the Tocaña does not share ancestry with the component that is more typical of the Llanos in Bolivia, represented here by the neighboring population of the Surui from the Mato Grosso do Sul, Brazil. The genomic patterns described are similar whether the unmasked (optimum *K* = 8; Fig. 4a) or masked Native American datasets (optimum *K* = 7; Fig. 4b) are used.

### D-statistics

**Fig. 5:**
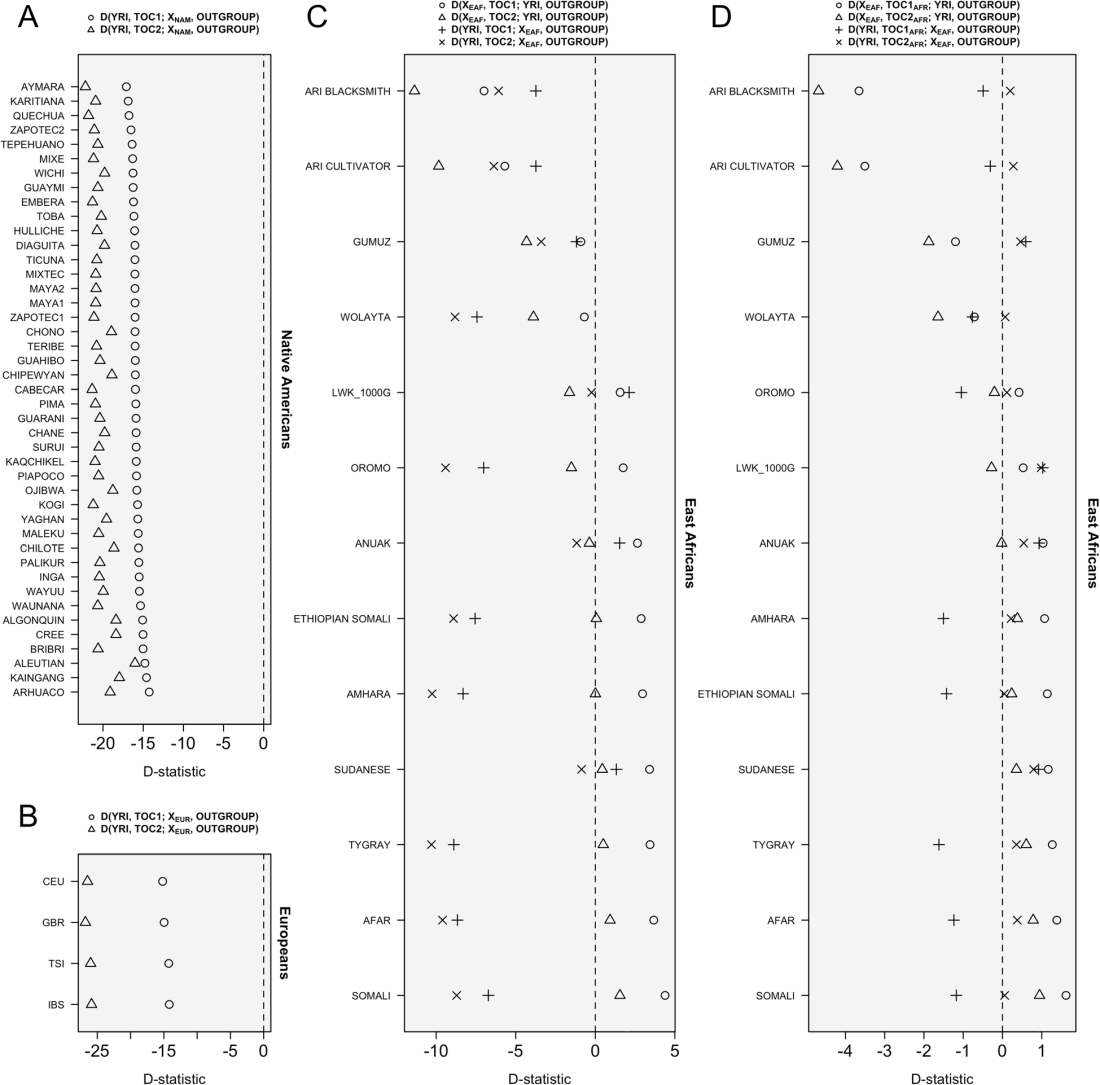
*D*-statistics computed on different population contexts and considering the two Tocaña individuals separately and Native American samples (**a**) European samples (**b**), East African samples (**c**), and East African samples but eliminating from the Tocaña genomes the non-African ancestry (**d**). Note that the values are not comparable between the three different figures; apart from using different scales the three analyses are based on different amounts of SNPs (see text for more information)

We computed *D*-statistics using different combinations of population datasets in order to formally test for the existence of different admixture components in the two Tocaña as suggested from admixture analysis. First, we were interested in testing if the Native American ancestry observed in the Tocaña was statistically significant. We therefore ran *D*(YRI, TOC_i_; *X*_*NA*_; Outgroup), with TOC*i* being each of the two Tocaña individuals, and *X*_*NA*_ the different Native American datasets used in the present study. *D*-statistics were highly significant (>98 K SNPs) for all the Native American datasets (Fig. 5a) and the two Tocaña. Interestingly, the highest significant value corresponded to the Aymara, followed by the Quechua (both from Bolivia).

We next computed *D*(YRI, TOC_i_; *X*_*EU*_; Outgroup), which used four different datasets as surrogate populations to test for European admixture in the two Tocañas (*X*_*EU*_). *D*-statistics were again highly significant (>573 K SNPs) for the four different datasets used; Fig. 5b.

Finally, we aimed to test for the presence of the East Africa component in the Tocaña as suggested by the ADMIXTURE analysis. We therefore computed *D*(YRI, TOC_i_; *X*_*EAF*_; Outgroup) and *D*(*X*_*EAF*_, TOC_i_; YRI; Outgroup), with *X*_*EAF*_ being the different East African datasets available. As shown in Fig. 5c, *D*-statistic was statistically significant (>148 K SNPs) for most of the population datasets from East Africa in both Bolivians (note that the threshold of significance was set to *D*-statistics < −2). This analysis was also very informative in showing that the Yoruba has a more important contribution to the Tocaña individuals than the East African populations, given that *D*-statistics showed more negative values when using East Africans as reference datasets; that is *D*(*X*_*EAF*_, TOC_i_; YRI; Outgroup). Also in good agreement with admixture proportions, *D*-statistics showed more negative values with #TOC2 than with #TOC1 in *D*(*X*_*EAF*_, TOC_i_; YRI; Outgroup) indicating a larger West African ancestry of #TOC2. Finally, ADMIXTURE analysis indicated that, out of the East African population samples, the Bantu ethnic group of Luhya from Kenya seemed to show some genetic affinities with the African component of the two Tocaña (Additional file 7); however, *D*-statistics were not significant for this dataset.

The fact that the two ‘Afro-Bolivians’ have a three-way continental admixture might distort the results of the *D*-statistics. In particular, the seeming contribution in the two Tocaña from East Africa could be due to an artifact provoked by the presence of the European ancestry in both, East Africa and in the two Tocaña. In fact, admixture analysis (Fig. 3) suggests the existence of such European component in both sets of populations. Therefore, in order to test further for this hypothesis, we repeated the *D*-statistic analyses by first eliminating the non-African component of the two Tocaña. For this analysis, we used the output from PCAdmix after filtering the non-African chromosomal segments. *D*-statistics carried out on these masked Afro-Bolivian genomes finally revealed a lack of statistically significant contribution of East Africa to the two Tocaña genomes (Fig. 5d).

### Dating the time of admixture

**Fig. 6:**
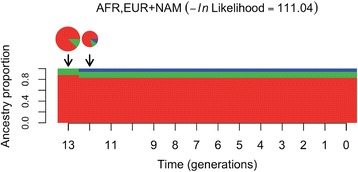
Time and model of admixture of ‘Afro-Bolivians’. The area of the pie charts above the migration model are proportional to the estimated number of migrants being introduced at each point in time (indicated by black arrows), as done in [35]

The output of PCAdmix (four haplotypes) was used to date the time of admixture as recorded in the two ‘Afro-Bolivian’ genomes and using the track length distribution of genomic segments inherited from different ancestral groups [17, 35]. The data indicate that the most likely scenario (−ln likelihood = 111.04) corresponds with a main admixture event occurring between a main African population (88.1 %) with a minor European population (11.9 %) about 13 generations ago; which correspond with 390 years ago (assuming 30 years per generation); Fig. 6. Right after (or even simultaneously), another admixture event would occur with the local Native American population, then configuring the main genome architecture of present-day ‘Afro-Bolivian’ genomes.

## Discussion and conclusions

The results of the present study indicate that the two ‘Afro-Bolivian’ genomes are a mosaic of different ancestries. These genomes have a main sub-Saharan component (~80 %), with a minor contribution (~20 %) coming from Europe and Native America.

The analyses suggest that West-Central Africa is the region that contributed the main proportion of African ancestry to ‘Afro-Bolivians’. The main signal comes from the Yoruba (Nigeria), but it seems also clear that there are other populations in West-Central Africa, mainly representing the Atlantic coast (e.g. Ghana, Senegal, Nigeria), that could also have contributed to the genome of the ‘Afro-Bolivians’ analyzed.

Stimulated by the recent findings of Heinz et al. [33] indicating the presence of mtDNA East African ancestry in the Yungas region, we tested for this ancestry in the autosomal genome of the two ‘Afro-Bolivians’ analyzed in the present study. In this regard, exploratory ADMIXTURE analysis suggested that the Luhya could, at least in part, explain the East African component observed in the Tocaña. Initial assessments derived from the analysis of *D*-statistics also indicated a seeming contribution of East African ancestry to the ‘Afro-Bolivian’ genomes. However, further analysis demonstrated that this signal could also be explained by computational artifacts provoked by the presence of European ancestry in the genome of the two Tocaña and in the East African populations.

While the African ancestry of ‘Afro-Bolivians’ constitutes a complex mosaic of African ancestries, their Native American component is probably more homogeneous, even though the analysis is very complicated by the admixed nature of all reference Native American populations [34]. The Native American component in the Tocaña genomes is compatible with a major contribution of the surrounding Aymara population (with which it is supposed to be admixed), and incompatible with admixture with populations that are more representative of the Llanos from Bolivia (characterized here by the neighboring Surui population).

The time of admixture, as reflected in the two ‘Afro-Bolivian’ genomes, could be derived from the tract length distribution of genomic segments inherited from distinct ancestries. The data indicate a main admixture event occurring at about 400 years ago between Africans, Europeans and Native Americans (Fig. 6). Interestingly, this event coincided with the period of maximum importation of slaves to Bolivia, which played a crucial role by compensating the diminishing indigenous labor force needed for the development of the National Mint of Potosí. The Mint of Potosí was the origin of most of the silver shipped to Spain at the time; the initial Mint dated to 1572, and the second factory was set in 1757 and was used till 1773.

Among the limitations of the present study is the fact that the reference populations in Africa, America and Europe, used in the present analyses may not represent the patterns of variability existing at the time of the TAST. In addition, although we compiled information on hundreds of individuals representing dozens of Native American and European populations, and more specially population samples in Africa, the size of the reference populations might still be small. In the future, an improved database could help corroborate the present findings and add more details on the demographic processes that led to the configuration of present-day ‘Afro-Bolivian’ genomic architecture (and ‘Afro-Americans’ in general).

With the arrival of new genotyping and computational technologies it is now possible to map with much better resolution the complex genomic mosaic of ‘Afro-Americans’. The case of ‘Afro-Bolivians’ is particularly interesting owing to their strong isolation from other African and Native American communities. Disentangling history of ‘Afro-Americans’ is hampered by the fact that many enslaved African people took unusual journeys after their enslavement. For example, Hans Jonathan, born as a slave on the Danish Caribbean island of St. Croix, was forced later in his life to migrate with his master to Copenhagen, Denmark, but when slavery was abolished in Denmark he was sentenced to go back as a slave to St. Croix. Jonathan escaped from slavery and finally landed in Iceland [36].

The present article shows that genomics is useful to reconstruct the individual ancestries of ‘Afro’ communities living in the Americas, accounting for the complex processes of admixture occurring since their arrival to the American continent. Most of the previous attempts were based on the analysis of uniparental markers [4, 6, 7, 9, 13, 14, 37, 38]. Although these markers are phylogeographically very informative at a population scale, they have limitations when dealing with individual ancestries (as it is the case of the two Tocaña individuals analyzed here), given that their mtDNA and Y-chromosome trace back to only two ancestors among the many thousands that could theoretically have contributed to their full genomes since the arrival of Europeans and the TAST into Bolivian territories.

## Methods

### Sampling

A collection of 105 saliva samples recruited from the Yunga’s region of Bolivia was previously characterized for mtDNA and a panel of Ancestry Informative Markers or AIMs [33]. A small subset of these samples was additionally analyzed for a selection of Y-chromosome markers [29]. For genome-wide analysis, we initially selected a subset of these samples (*n* = 9) that showed a large membership of African ancestry in the analysis of a small panel of AIMs. We obtained written informed consent for all the donors prior the research, which includes consent for publication individual data. Rights of participants were safeguarded during the research and their identity was protected. The study was approved by the ethical commission of the Universidade de Santiago de Compostela (Galicia; Spain) which depends on Ethics Committee of Galicia (Spain) and by the Ethics Committee of Universidad Mayor, Real y Pontificia de San Francisco Xabier de Chuquisaca (Bolivia). The study conforms with all applicable Spanish normative, this is to say, Law for Biomedical Research (Law 14/2007–3 of July), Law 41/2002 of Autonomy of the Patient, Decree SAS/3470/2009 for observational studies and Law 15/1999 of Data Protection.

### Genotyping

Samples were genotyped using the Axiom® Genome-Wide Human Origins 1 Arrays at the Centro Nacional de Genotipado (CEGEN) of Santiago de Compostela (Spain). After processing the samples, the Affymetrix Genotyping Console™ (GTC) 4.1.2 Software package was used to generate QC metrics and genotype calls following commercial recommendations. This procedure includes generation of sample dish QC (DQC) values. Samples with a DQC value lower than the default DQC threshold of 0.82 were dropped from the study. The “AxiomGT1_all” algorithm was used for genotyping this group of high quality samples using all SNPs on the array. Unfortunately, not all the samples initially selected for genotyping contained enough DNA (and/or of the required quality) for genome-wide SNP genotyping. Finally, only the analysis of two of the samples succeeded, with a total of 580,144 SNPs genotyped. These two samples have been recruited in the locality of Tocaña, and are referred to here as #TOC1 and #TOC2 (original sample ID: #Tocana301 and #T303; respectively).

### Reference populations

We intersected the SNP data obtained from the two ‘Afro-Bolivians’ with genome population data downloaded and processed from different SNP repositories; Additional file 1. Comparative population analyses were carried out using different sets of data. The different combination of populations sets used for each analysis is also indicated in Additional file 1.

Data from The 1000 Genomes Project (hereafter 1000G) provides the dataset with the largest SNP overlap with the Tocaña data. The data from 1000G was retrieved from the original repository as done in Pardo-Seco et al. [39]; these analyses involved 580,144 SNPs (1.7 % of missing data).

The lowest SNP overlap occurred when intersecting the Tocaña SNP set with a large compilation of population samples that includes 1,161 Africans (representing 57 different populations/ethnic groups); these analyses involved 25,192 SNPs (0.7 % of missing data).

Some *ad hoc* analyses were carried out using selected sub-sets of populations with the aim to increase the number of SNPs (specific numbers are given in the main text where relevant). For instance, analyses of the Native American component observed in the two Tocaña individuals were carried out using the unmasked (0.2 missing data) and masked (7.8 % missing data) dataset from Reich et al. [34]; these datasets include 99,378 and 92,619 SNPs, respectively.

### Statistical analysis

PLINK [40] was used to compute IBSe values from SNP data. Before proceeding with all population analyses, we tested the two Tocaña genomic profiles for potential close relationships given that both individuals were sampled in the same location. We followed the procedure detailed in Gómez-Carballa et al. [41]. Identity-by-descent (IBD) values for this pair of genetic profiles are not compatible with close familial relationships after based on statistical tests proposed before [42, 43]; *P*-value = 1.

Admixture components of the two Bolivian individuals were performed using the ADMIXTURE software [25], which uses a maximum likelihood estimation of individual ancestries from multi-locus SNP data.

Local ancestry assignment of ancestry-specific haplotypes across the genome was carried out using PCAdmix 1.0 [44]. PCAdmix requires haplotype data; therefore, in order to prepare input files for PCAdmix, SNP unphased data were imputed and haplotypes were built using Beagle 3.3.2. [45]. For this particular analysis, we selected populations from 1000G complemented with some Native American groups analyzed in Reich et al. [34] in order to represent the three main ancestral groups of the Tocaña: Yoruba and Luhya (representing African ancestry), CEU (representing European ancestry), and Aymara, Guahibo, Guarani, Hullicje Maya, Mixe, Piapoco, Quechua, Ticuna, Toba, and Wichi (representing Native American ancestry). We assigned genomic segments to the three possible ancestries following a posterior probability threshold of 0.8.

In order to discriminate among clusters of genetic variation in the population sets analyzed, multidimensional scaling (MDS) was carried out on a matrix of pairwise individual IBS values. MDS was performed using the function *cmdscale* (library *stats*) from R (http://www.r-project.org). PCAmask [17] was additionally carried out on genomic segments assigned to Native American, African, or European ancestry. This analysis uses a haplotype-based algorithm and therefore includes segments of the genome that are heterozygous for ancestry (thus minimizing missing data). Given that PCAmask uses the output of PCAdmix, we run PCAmask using the same sub-set of samples.

We additionally computed the four-population test, implemented as *D*-statistics [46], in order to determine the relationship between the two Tocaña SNP profiles individually *vs*. different population sets [34, 47–49]. This is a formal test for admixture that measure allele frequency correlations among populations; it provides statistical evidence of admixture and information on the directionality of the gene flow. *D*-statistic was computed using the weighted block jackknife procedure (block size of 5 MB) [47].

For the computation of *D*-statistics, we built an outgroup that is symmetrically related to all modern human population groups, by creating an individual profile possessing the ancestral alleles at all sites. The use of this artificial outgroup allowed us to infer *D*-statistics, thus ensuring that there is no differential gene flow between the outgroup and the population sets used; this is particularly important in our population context given that the three main continental ancestries are present in our sample sets. The construction of this outgroup involved the following steps. First, an auxiliary ancestral sample VCF file was generated containing all the ancestral alleles as alternative alleles. Second, all the chromosome sites from 1000G VCF files where parsed looking for “AA” codes, the ancestral alleles where detected and the alternative alleles where substituted with the ancestral alleles. Only the first four VCF columns where kept, then the ancestral allele was added, and the rest of the columns where filled with single dots. The genotype column was filled with reference homozygous (0/0) in cases when only the reference was reported, and with alternative heterozygous (0/1) when an ancestral allele different from the reference was reported.

Finally, timing of admixture was estimated by way of analyzing the tract length of chromosomal segments attributed to different ancestries, using the software *Tracts* [35]; see also Moreno-Estrada et al. [17].

## Additional files

Additional file 1: Population datasets used in the present study. (XLS 66 kb) Additional file 2: Average IBS values between the two individuals from Tocaña and individuals from various continental regions represented in (A) 1000G, and (B) a large dataset of African regions. (TIFF 10002 kb) Additional file 3: MDS of the two Tocaña individuals *vs*. the population sets from 1000G representing the main continental groups. See Additional file 1 for more information on population datasets. (TIFF 2502 kb) Additional file 4: MDS of Tocaña against 57 datasets representing different sub-continental African regions. One population of European ancestry (CEU) from 1000G were used for reference. See Additional file 1 for more information on population datasets. (TIFF 27503 kb) Additional file 5: Analysis of admixture as in Fig. 3a for additional *K* values. (TIFF 15628 kb) Additional file 6: Ancestry memberships as computed using the unsupervised clustering algorithm from ADMIXTURE using the 1000G datasets and the Tocaña, for an optimum value of *K* = 5. (XLSX 57 kb) Additional file 7: Ancestry memberships as computed using the unsupervised clustering algorithm from ADMIXTURE and a large dataset representing main sub-continental African regions and the Tocaña, for an optimum value of *K* = 12. (XLSX 14 kb) Additional file 8: Analysis of admixture as in Fig. 3b for additional *K* values. (TIFF 31254 kb)
