# Supplementary figures and images for: Mapping the genomic mosaic of two ‘Afro-Bolivians’ from the isolated Yungas valleys
Source: BMC Genomics. 2016 Mar 9;17:207. doi: 10.1186/s12864-016-2520-x (PMC4784306; doi:10.1186/s12864-016-2520-x)

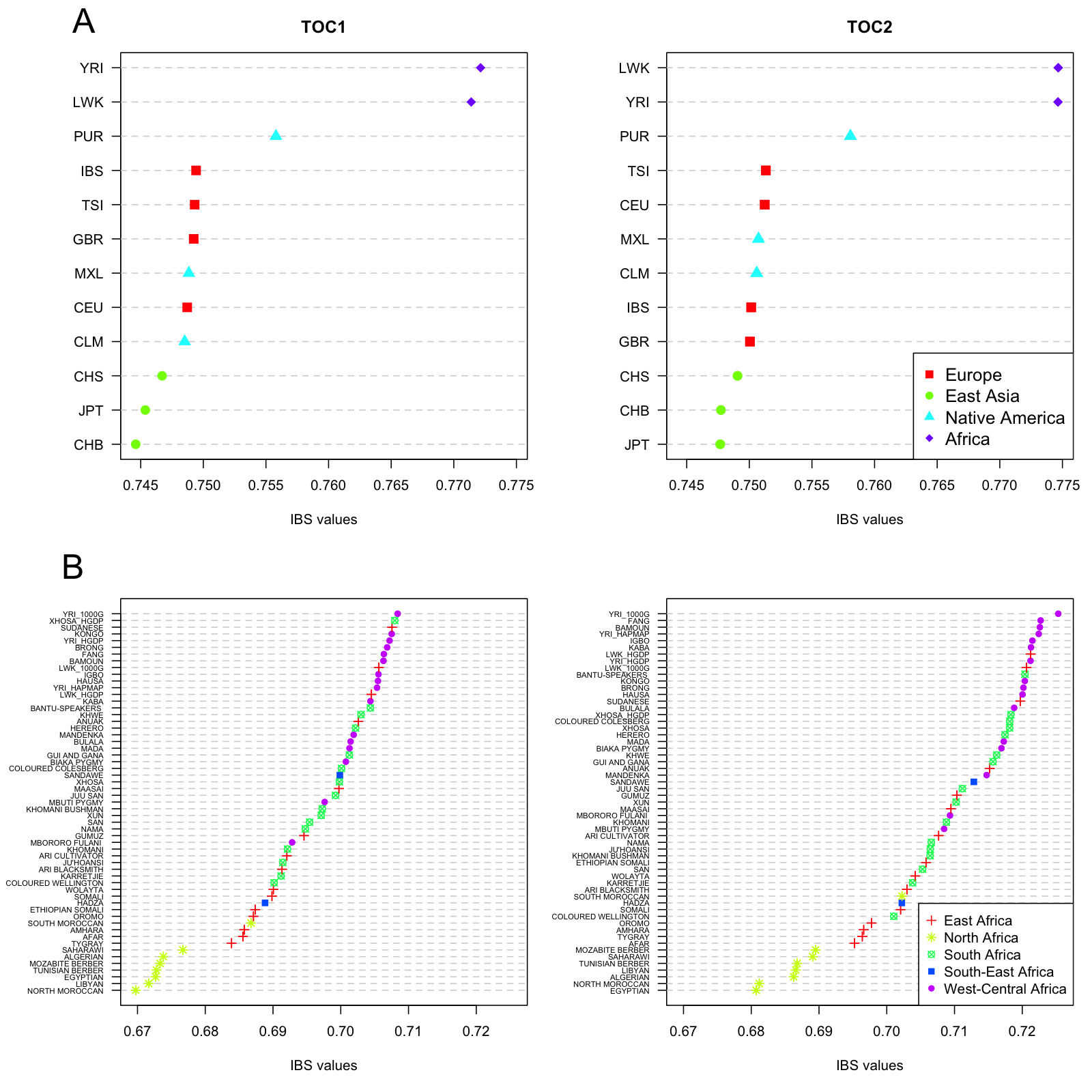

Supplement: Additional file 2: — Average IBS values between the two individuals from Tocaña and individuals from various continental regions represented in (A) 1000G, and (B) a large dataset of African regions. (TIFF 10002 kb) [file 12864_2016_2520_MOESM2_ESM.tiff]

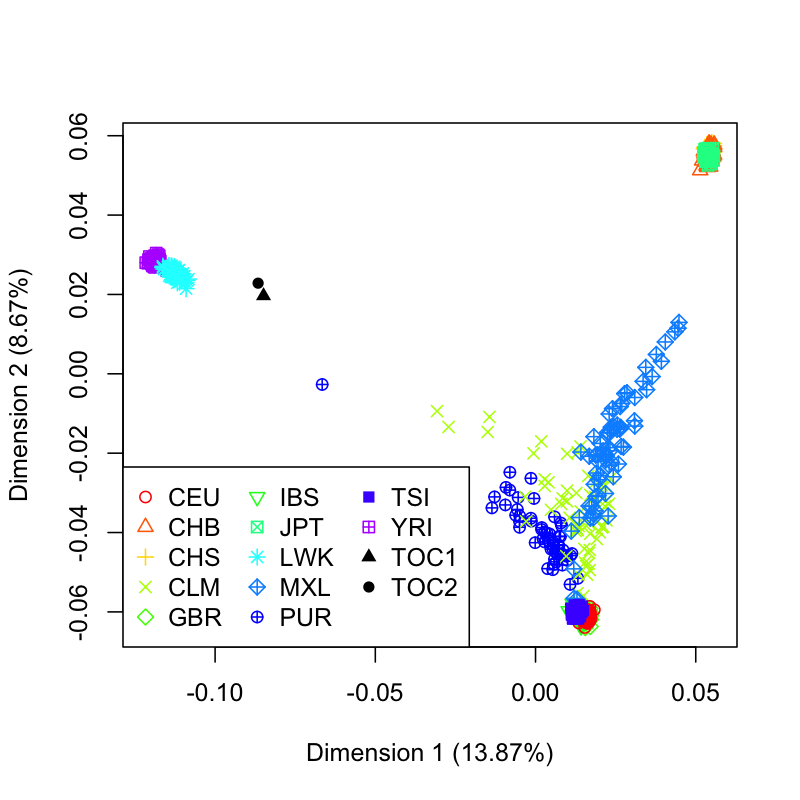

Supplement: Additional file 3: — MDS of the two Tocaña individuals vs. the population sets from 1000G representing the main continental groups. See Additional file 1 for more information on population datasets. (TIFF 2502 kb) [file 12864_2016_2520_MOESM3_ESM.tiff]

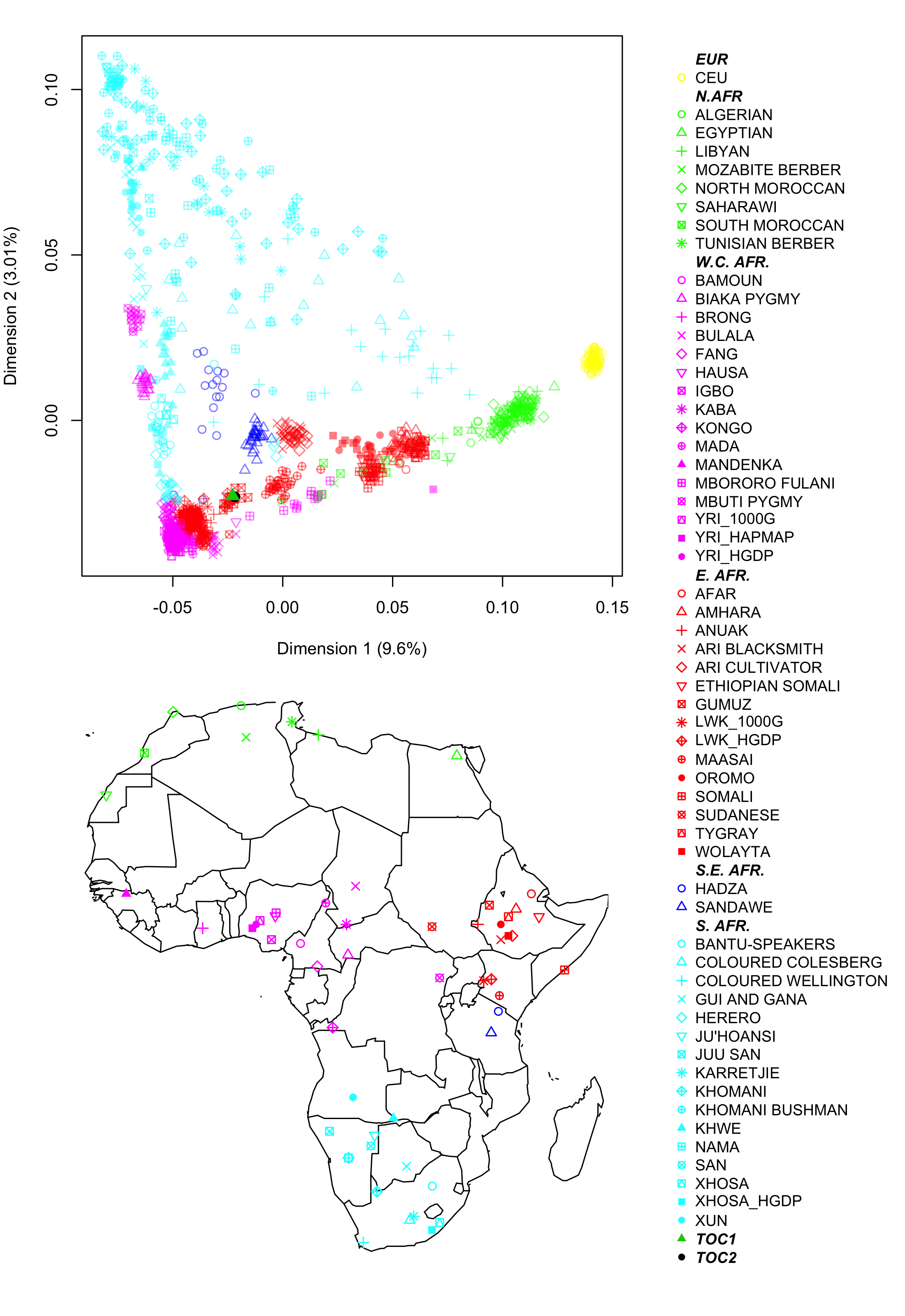

Supplement: Additional file 4: — MDS of Tocaña against 57 datasets representing different sub-continental African regions. One population of European ancestry (CEU) from 1000G were used for reference. See Additional file 1 for more information on population datasets. (TIFF 27503 kb) [file 12864_2016_2520_MOESM4_ESM.tiff]

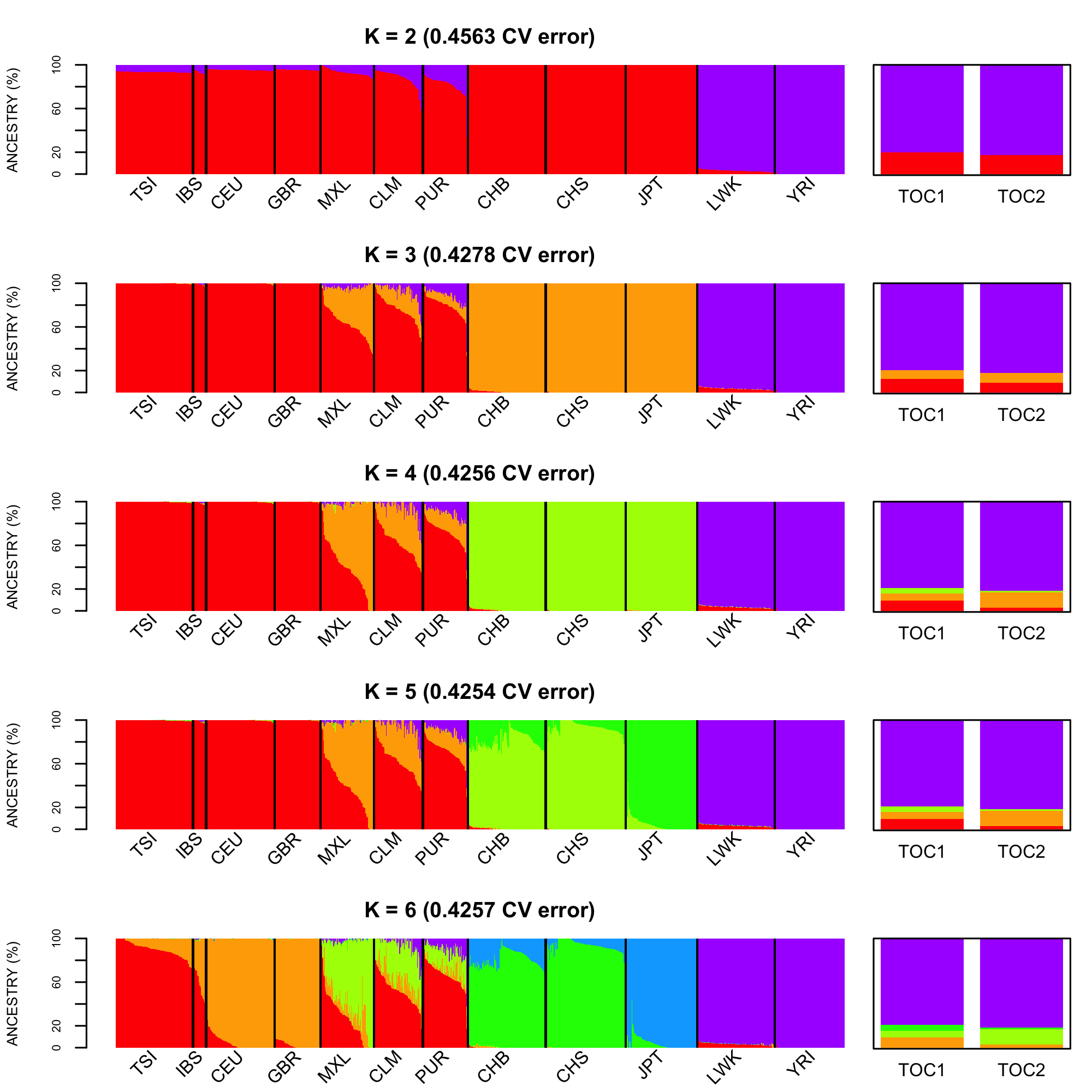

Supplement: Additional file 5: — Analysis of admixture as in Fig. 3a for additional K values. (TIFF 15628 kb) [file 12864_2016_2520_MOESM5_ESM.tiff]

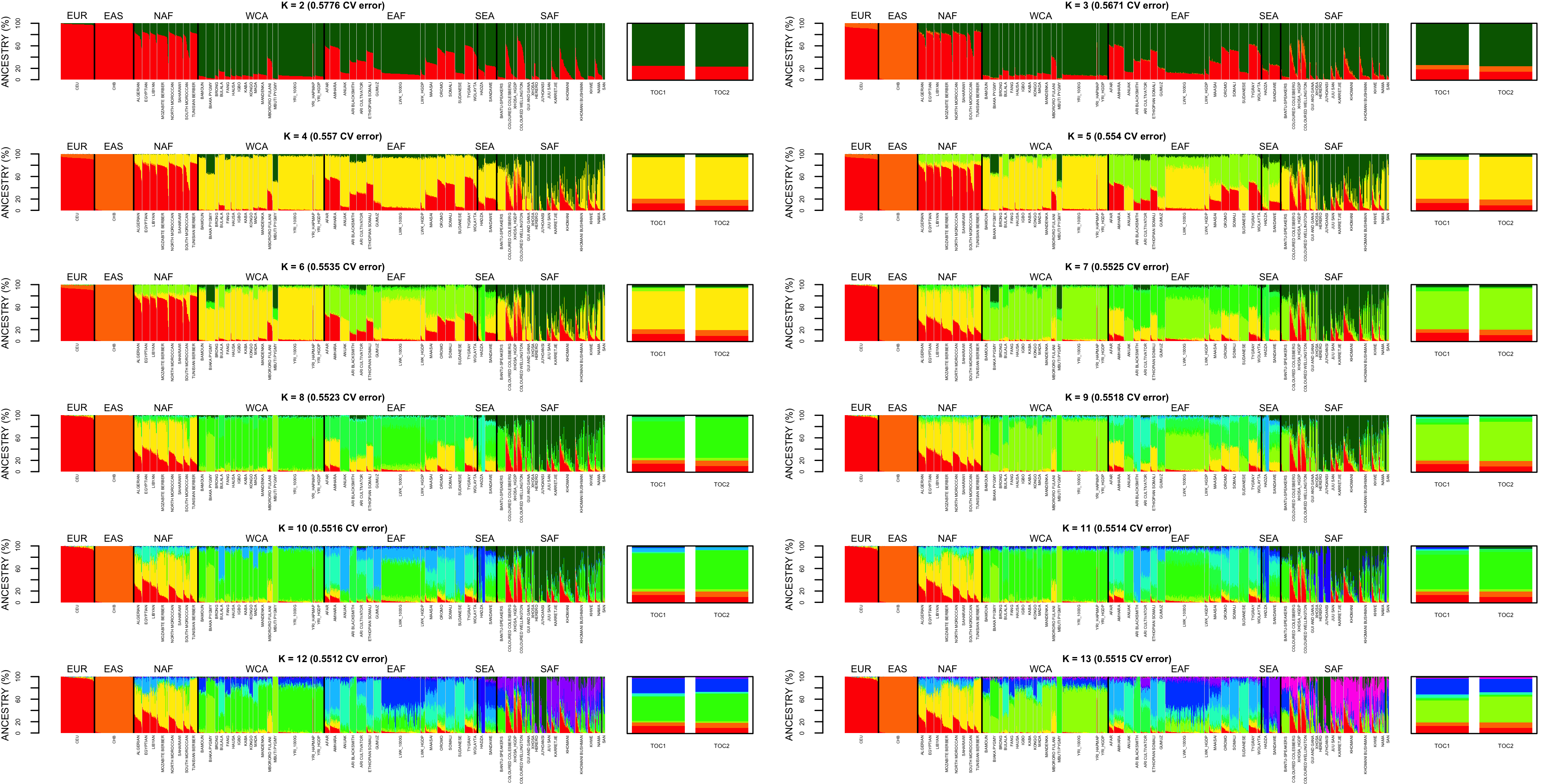

Supplement: Additional file 8: — Analysis of admixture as in Fig. 3b for additional K values. (TIFF 31254 kb) [file 12864_2016_2520_MOESM8_ESM.tiff]
